# Non-Coding RNAs in Lung Cancer: Contribution of Bioinformatics Analysis to the Development of Non-Invasive Diagnostic Tools

**DOI:** 10.3390/genes8010008

**Published:** 2016-12-26

**Authors:** Meik Kunz, Beat Wolf, Harald Schulze, David Atlan, Thorsten Walles, Heike Walles, Thomas Dandekar

**Affiliations:** 1 Functional Genomics and Systems Biology Group, Department of Bioinformatics, Biocenter, University of Wuerzburg, 97074 Wuerzburg, Germany; meik.kunz@uni-wuerzburg.de (M.K.); beat.wolf@hefr.ch (B.W.); 2 University of Applied Sciences and Arts of Western Switzerland, Perolles 80, 1700 Fribourg, Switzerland; 3 Institute of Experimental Biomedicine, University Hospital Wuerzburg, 97080 Wuerzburg, Germany; schulze_h@ukw.de; 4 Phenosystems SA, 137 Rue de Tubize, 1440 Braine le Château, Belgium; phenosystems@gmail.com; 5 Department of Cardiothoracic Surgery, University Hospital of Wuerzburg, 97080 Wuerzburg, Germany; walles_t@ukw.de; 6 Department of Tissue Engineering and Regenerative Medicine, University Hospital Wuerzburg, Roentgenring 11, 97070 Wuerzburg, Germany; heike.walles@igb.fraunhofer.de; 7 Translational Center Wuerzburg “Regenerative therapies in oncology and musculoskeletal disease” Wuerzburg branch of the Fraunhofer Institute Interfacial Engineering and Biotechnology (IGB), Roentgenring 11, 97070 Wuerzburg, Germany; 8 BioComputing Unit, European Molecular Biology Laboratory (EMBL) Heidelberg, Meyerhofstraße 1, 69117 Heidelberg, Germany

**Keywords:** lung cancer, non-invasive biomarkers, miRNAs, lncRNAs, early diagnosis, bioinformatics, algorithm

## Abstract

Lung cancer is currently the leading cause of cancer related mortality due to late diagnosis and limited treatment intervention. Non-coding RNAs are not translated into proteins and have emerged as fundamental regulators of gene expression. Recent studies reported that microRNAs and long non-coding RNAs are involved in lung cancer development and progression. Moreover, they appear as new promising non-invasive biomarkers for early lung cancer diagnosis. Here, we highlight their potential as biomarker in lung cancer and present how bioinformatics can contribute to the development of non-invasive diagnostic tools. For this, we discuss several bioinformatics algorithms and software tools for a comprehensive understanding and functional characterization of microRNAs and long non-coding RNAs.

## 1. Introduction

Lung cancer is the main cause of cancer-associated mortality worldwide. Non-small cell lung cancer (NSCLC) with the two major pathologic types adenocarcinoma (AC) and squamous cell carcinoma (SQ) [1,2] represents with 85% the most often diagnosed subtype [3,4], whereas small-cell lung carcinoma (SCLC, 15%) is the most aggressive subtype but less observed [5]. Several studies reported differences in the gene expression characteristics between AC and SQ, but also between both lung cancer subtypes [1,5,6,7,8]. Most prominently altered molecular marker genes in SCLC are associated with neuronal differentiation and/or growth such as the human achaete-scute homolog 1 (ASCL1) and the glycine receptor alpha1 subunit gene (GLRA1) [8,9], whereas NSCLC is characterized by deregulation of the Epidermal growth factor receptor (EGFR) and tumor-suppressor p53 [10,11].

About 25%–30% of patients are at limited stage of disease at time of diagnosis warranting surgical therapy. The remaining majority of patients depends on systemic therapy. Lung cancer detection is often too late due to missing patient symptoms at early disease and the lack of accurate diagnostic tools [12]. Notably, diagnosis at early stages can increase patient survival rate up to 45%–53% [12]. Diagnosis currently includes computer tomography (CT) or PET-CT scan which have a high false-positive rate (fall-out rate) up to 28% (incorrect as lung cancer identified individuals which are healthy, resulting in unnecessary additional diagnostics), followed by invasive biopsy and bronchoscopy [12,13,14]. Systemic therapy is based on platinum-based chemotherapy that can be combined with targeted therapies (e.g., tyrosine kinase inhibitor gefitinib) according to the biomarker status of the tumor. Targeted therapies raise hope for treatment improvements, but unfortunately lead commonly to resistance development after several months [11]. The five-year survival rate in these patients is less than 5%.

Over the past decades, diagnostics have mainly focused on proteins which represent only a small part of the entire human genome [12,15]. For example, the serum markers Cytokeratin 19 Fragment, Cancer Antigen 125, Cancer Antigen 19-9, tissue polypeptide antigen and Neuron-specific Enolase were extensively investigated [12,16,17,18,19]. However, due to the high false positives and low sensitivity, none of them can currently serve as stand-alone biomarker for early lung cancer detection [12,20,21]. Thus, accurate and innovative diagnostic biomarker tests for early disease detection are required. Ideally, for potential clinical application such tests should have the following characteristics: they should be minimal invasive, easily accessible in body fluids and highly specific, sensitive and robust [15,22].

Transcriptome analysis revealed that non-coding RNAs (ncRNAs; no protein encoding) such as microRNAs (miRNAs) and long non-coding RNAs (lncRNAs) represent more than 90% of the transcribed human genome [15,23,24,25]. Recent studies demonstrated that miRNAs and lncRNAs regulate several targets and are associated with important biological processes and signaling pathways, also often show a tissue specific expression [26,27,28]. Moreover, several miRNAs and lncRNAs were found to play an important role in lung cancer pathogenesis and can function as molecular marker for cancer metastasis and prognosis [15,29,30,31]. Thus, investigating the functional role of miRNAs and lncRNAs can lead to a better understanding of lung cancer tumorigenesis which opens new windows for effective diagnostic strategies for a better clinical management of lung cancer.

In this review, we present how bioinformatics can contribute to the development of non-invasive diagnostic tools, in particular we focus on lung cancer as an example disease. We report the current state of research of miRNAs and lncRNAs as novel diagnostic biomarkers and discuss bioinformatics algorithm and software tools for understanding the functional role of miRNAs and lncRNAs. However, all presented bioinformatics analysis tools can also be applied to other tumors and, where appropriate, to other diseases.

## 2. miRNAs as Diagnostic Biomarkers

**Figure 1:**
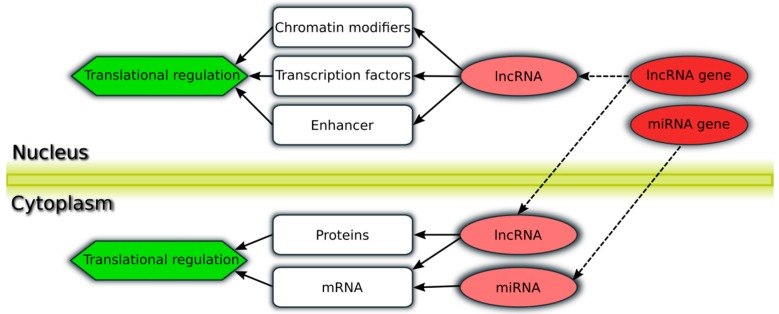
Schematic overview of miRNA and lncRNA regulation. miRNAs are transcribed as miRNA gene (light red circle) in the nucleus and further transported (dashed arrows) in the cytoplasm (darker red circle) where they regulate (continuous arrows) translation (green hexagon) through complementary mRNA binding (white rectangle). lncRNAs are transcribed in the nucleus (light red circle) and not only transported (dashed arrows) in the cytoplasm (darker red circle) and influence translation, e.g., through mRNA and protein binding (white rectangles), but can also regulate transcription (green hexagon), e.g., through chromatin modifier binding (white rectangles) in the nucleus.

miRNAs are small ncRNAs (~22 nucleotides) which are highly conserved among different organism. They lie in intronic or intergenic regions of coding and non-coding genes. miRNAs regulate gene expression in the cytoplasm through binding with their conserved seed-region to messenger RNA (mRNA) in a sequence-specific manner, resulting in mRNA degradation or inhibition which leads to a reduced translational activity or inhibition of translation [30,32,33,34,35,36] (see Figure 1).

miRNAs such as miRNA-21, let-7, miRNA-145, miRNA-155 and miRNA-210 are known to have an implication in lung tumorigenesis [37,38,39,40,41]. They are reported to show altered expression levels in lung cancer and correlated with tumor stage and patient survival [42,43,44]. Moreover, several miRNAs show a co-expression in lung cancer patient, e.g., miRNA-21, miRNA-145 and miRNA-155 or let-7g and miRNA-21 [37,45]. Table 1 summarizes different clinical miRNA lung cancer studies including background information (number of patient, tumor stage and histological subtype; if available).

Interestingly, miRNAs can also distinguish between the two lung cancer subtypes NSCLC and SCLC. For example, Du et al. (2010) [5] identified 41 differentially expressed miRNAs that effective distinguish both subtypes, for instance miRNA-17-5p, miRNA-135, miRNA-103, miRNA-107, miRNA-301 and miRNA-338 were over-expressed in SCLC relative to NSCLC cells and have a potential to serve as potential biomarker in SCLC diagnosis. More interestingly, they observed that the known NSCLC associated miRNAs miRNA-29a/b/c, miRNA-24 and the oncogenic miRNA-21 and miRNA-221/222 are significantly down-regulated in SCLC. Similarly, Lee et al. (2011) reported that the expression of the seven miRNAs miRNA-21, miRNA-29b, miRNA-34a/b/c, miRNA-155 and let-7a are not related to SCLC patients [46].

Beside them, studies reported that miRNA signatures can differentiate the histological types of NSCLC. Lebanony et al. (2009) [47] identified miRNA-205 as highly specific marker for SQ to clearly distinguish it from AC which was independently validated by Bishop et al. (2010) [48] using resected NSCLC biopsies. Moreover, Landi et al. (2010) [30] identified that miRNA expression profiles significantly differentiated AC from SQ in which an altered expression of five miRNAs (miRNA-25, miRNA-34c-5p, miRNA-191, let-7e and miRNA-34a) strongly predicted survival for SQ.

As altered miRNA expression levels do not only correlate with patient relapse and survival but can also clearly distinguish between lung cancers, thus they might function as novel diagnostic biomarkers.

Several studies highlight miRNA expression signatures as non-invasive diagnostic tools for early detection and classification of lung cancer including first promising clinical results. For example, Hennessey et al. (2012) [49] reported from a phase I/II biomarker study the potential of a serum miRNA-15b/miRNA-27b pair as sensitive and effective biomarkers for the early NSCLC detection. Moreover, Sozzi et al. (2014) [13] described the clinical utility of blood based miRNA signatures which have a high potential to reduce false-positive rates of low dose CT (LDCT) screenings (3.7% compared to 19.7% for LDCT alone) as an additional diagnostic tool (MILD-trial). Similarly, Montani et al. (2015) developed the blood miRNA-test for identification of optimal patients for LDCT screening (COSMOS-trial; accuracy, sensitivity and specificity approximately 75%–78%) [14].

In the context of having been mainly developed for smokers as a first-line tool for LDCT screenings with *per se* high false-positive rates, these miRNA blood tests reduce unnecessary LDCT screenings in disease-free individuals of such high-risk cohorts. In case of a general population such tests appear probably not yet very beneficial and need further improvements and validation [13,14,49].

On the other hand, studies investigating the potential of miRNAs to differentiate between NSCLC patients and smokers have been started. For example, Zhu et al. (2016) [40] validated the diagnostic value of a four serum miRNA signature (miRNA-182, miRNA-183, miRNA-210 and miRNA-126) for early diagnosis which can also distinguish NSCLC patients from current smokers. This points to the usage of a combined biomarker signature for a better diagnostic value and an effective molecular characterization of lung cancer patients [37,45].

Nevertheless, miRNA based signatures have drawbacks and limitations: They depend on technical accuracy and proper validation, standardization and normalization schemes. miRNAs were detected either in tumor tissue or blood samples. However, miRNA levels are different in plasma and serum, and the overlap of miRNAs present both in blood and tissue is small [14,50]. This points out the challenge of miRNA signatures as a clinically reliable blood test. However, given the low overlap of miRNAs between different samples, there exists a common consensus of known miRNAs expressed in lung cancer, e.g., miRNA-21 and/or miRNA-30d, in which one of them is at least found across different studies and/or known to be expressed in tissue, plasma and serum [13,44,49,50,51]. Such statistically significantly expressed miRNAs were also independently validated by bioinformatics meta-analyses with miRNA-21 and miRNA-210 as most consistently reported miRNAs in lung cancer [52,53]. Thus, circulating blood signatures based on those miRNAs represent a high potential as an effective diagnostic tool for early lung cancer detection.

## 3. lncRNAs as Diagnostic Biomarkers

lncRNAs represent a large and diverse class of ncRNAs that play an important biological role. They are multi-exonic transcripts greater than 200 nucleotides and often less conserved among species [7,28,54,55]. lncRNAs show altered expression and regulate important biological processes and pathways associated with lung cancer pathogenesis and progression [15]. They have a complex regulatory effect ranging from transcriptional regulation (enhancer, chromatin modifier and transcription factor (TF) binding) and regulation of epigenetic processes (nuclear lncRNAs) to translational regulation through mRNA and protein binding as well as miRNA sponging (cytoplasm lncRNAs) [54,55,56,57,58] (see Figure 1). Thus, functional characterization is challenging and most lncRNAs are not well understood: currently, several thousand lncRNA transcripts have been annotated, however the majority is functionally uncharacterized [59,60,61,62].

As lncRNAs show a tissue- and disease-specific expression they emerged as potential diagnostic biomarkers [2,7]. lncRNAs are already known as promising biomarkers for different diseases, e.g., LIPCAR as biomarker for cardiac remodeling [63] or HULC for liver cancer [15,64]. Moreover, lncRNA based diagnostic tests such as the lncRNA prostate cancer antigen 3 (PCA3) as urinary biomarker for prostate cancer are already available for clinical use [65].

Therefore, lncRNAs are currently also under investigation for lung cancer, showing high potential for developing effective screening tests for diagnosis [66,67]. Table 2 summarizes different clinical lncRNA lung cancer studies including background information (number of patient, tumor stage, histological subtype; if available).

The most promising lncRNA candidate is the metastasis-associated lung AC transcript 1 (MALAT1) which is highly expressed e.g., in lung and pancreas. MALAT1 is functionally well-characterized and known to be a prognostic marker for early-stage NSCLC as well as cancer metastasis [29,68]. Moreover, MALAT1 has been shown to serve as blood-based biomarker for the early detection of NSCLC. For example, Yao et al. (2012) identified a four serum biomarker signature containing MALAT1 and three proteins that show a high diagnostic accuracy for detecting early stage NSCLC [12]. Similarly, Weber et al. (2013) reported MALAT1 as non-invasive and effective diagnostic biomarker for NSCLC diagnosis [22]. As the authors observed a low sensitivity, they suggest that MALAT1 might not be a single biomarker but applicable as a complementary biomarker [22]. The lung AC-specific lncRNA colon cancer-associated transcript 2 (CCAT2) displays altered expression, promotes invasion of NSCLC and can serve as a biomarker for lymph node metastasis [69]. The lncRNA HOX transcript antisense intergenic RNA (HOTAIR) functions as gene expression repressor through recruitment of chromatin modifiers and correlates with metastasis and poor prognosis in NSCLC [71,77].

Several lncRNAs show a co-expression in lung cancer patients. For example, Yang et al. (2013) validated the differential expression of 8 mRNAs, 8 lncRNAs and 5 miRNAs in NSCLC cells, in which co-expression between genes (e.g., FN1, CTSB, EGFR and NKD2), lncRNAs (e.g., BX648420, ENST00000366408 and AK126698) and miRNAs (e.g., miRNA-26a and let-7i) were identified, also playing a potential key role in cisplatin resistance [72]. More recently, Sui et al. (2016) confirmed a correlation between AFAP1-AS1 and LINC00472 and clinical features in AC patients [73].

Further lncRNAs known to be associated with different cancer types are also under investigation as diagnostic biomarker in lung cancer. For instance, Tantai et al. (2015) showed that the lncRNAs XIST and HIF1A-AS1 have a significantly increased level in tumor tissues or serum from NSCLC patients, highlighting a clinical significance as effective diagnostic screening for NSCLC when combining both lncRNAs [74].

lncRNAs were also investigated regarding their potential as clinical biomarkers to predict lung cancer risk and treatment response. In this context, studies of Gong et al. (2016) found that genetic polymorphisms of well-characterized lncRNAs such as CCAT2, HOTAIR and MALAT1 were significantly associated with lung cancer susceptibility and platinum-based chemotherapy response, indicating that they might function as clinical biomarkers [75]. Furthermore, Yuan et al. (2016) found in a large-scale meta-analysis of 690,564 single-nucleotide polymorphism (SNPs) in 15,531 autosomal lncRNAs a genetic SNP risk locus (1p31.1) in the lncRNA NEXN-AS1 which influence the secondary structure and is statistically associated with lung cancer risk [76]. It could thus serve as potential risk biomarker for lung cancer diagnosis. The potential of lncRNAs as diagnostic biomarkers was also confirmed by several meta-analyses with MALAT1 and the human urothelial carcinoma associated 1 (UCA1) as most promising candidates in lung cancer patient [29,78,79,80].

In addition, altered lncRNA expression levels might also accurately distinguish between AC and SQ and predict the clinical outcome for both NSCLC subtypes [1,2,6,7]. White et al. (2014) characterized 567 RNA-Seq datasets from AC and SQ tumors and found 463 and 315 up- and down-regulated lncRNA, respectively, in AC tumors relative to SQ. Moreover, they reported that 27 lncRNAs were differentially expressed between AC and SQ that can potentially serve as important biomarkers for lung cancer diagnosis [7]. Furthermore, studies of Zhang et al. (2015) identified 1646 differentially expressed lncRNA transcripts, in which the lncRNA LINC01133 showed the largest up-regulation in SQ but not in the AC samples and correlates with shorter survival time [2]. Recently, Wei et al. (2016) reported four six-lncRNA signature patterns that are significantly associated with AC and SQ patient survival [1]. More interestingly, the authors also demonstrated that knockdown of the up-regulated lncRNA AFAP1-AS1 and LINC00511 impaired AC cell proliferation, while knockdown of PVT1 inhibited SQ cell growth [1].

However, to the best of our knowledge, currently no study has been directly carried out to identify lncRNA expression signatures that can differentiate NSCLC and SCLC lung cancer subtypes. An explanation might be that investigating lncRNAs in lung cancer is a relatively new research field and therefore only very few lncRNAs are well-characterized. Moreover, lncRNA studies focused primarily on NSCLC as the more commonly diagnosed subtype, but also mainly on lncRNAs that were already reported from other cancer types, e.g., the colon cancer lncRNA CCAT2. However, there exist reports on lncRNA characterization in different lung cancer subtypes. For example, Qiu et al. (2014) identified that the lncRNA CCAT2 shows a specific expression in AC and might function as biomarker for lymph node metastasis [69], whereas Chen et al. (2016) recently found that CCAT2 serves also as an independent unfavorable prognostic factor in SCLC patients [70]. In this regard, it would be of high importance for the future to investigate differentially expressed lncRNAs between NSCLC and SCLC in order to develop a diagnostic biomarker signature that can accurately distinguish between both lung cancer subtypes.

## 4. Bioinformatics Databases, Algorithms and Analysis Tools

The number of annotated miRNAs and lncRNAs is growing dramatically, however experimental characterization is challenging [15,59,81,82]. Bioinformatics analysis can contribute to a comprehensive functional understanding of miRNAs and lncRNAs. Instead of discussing experimental methods (several experimental methods are reviewed in [83]), in this section we will discuss several databases and bioinformatics algorithms. A summary of them is given in Table 3, Table 4 and Table 5. It is worth mentioning that all the presented databases and tools are also be applicable to other tumors and diseases.

### 4.1. Bioinformatics Databases

**Figure 2:**
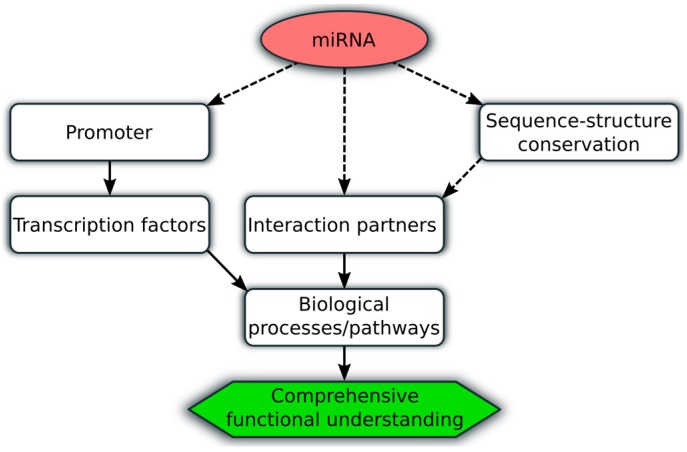
Workflow for integrated bioinformatics functional analysis of miRNAs and lncRNAs. Illustration of integrated bioinformatics analysis of ncRNAs (miRNAs, lncRNAs; red circle) which should focus on the sequence, structure, promoter and interaction partner prediction combined with functional analysis (rectangles). Dashed arrows represent the three main analysis steps (e.g., promoter analysis), whereas continuous arrows show the subsequently functional analysis step using the obtained results from the previous steps (e.g., transcription factors) to get a comprehensive functional understanding of ncRNAs (green hexagon).

Popular databases such as Rfam [84] represent information about several RNA families including sequence and consensus secondary structure information, whereas LNCipedia [59], LncRBase [60] and miRBase [109] provide information about specific families including further information, e.g., about experimental data, tissue expression and targets. On the other hand, databases such as MiR2Disease [110] and LncRNADisease [114] focus on the disease and interaction specific context based on the literature and/or experimentally disease data. All these databases provide information about miRNAs and lncRNAs and allow a fast overview regarding their sequence, structure and functional role. However, most miRNAs and lncRNAs are newly detected without concrete knowledge about their functional role which requires integrated bioinformatics analysis for comprehensive understanding. In this context, they should combine phylogenetic sequence-structure conservation analysis with functional interaction partner, biological process and pathway as well as promoter analysis (see Figure 2).

### 4.2. Phylogenetic Sequence-Structure Conservation

Sequence data are available from the Ensembl (http://www.ensembl.org/) and UCSC genome browser. The sequence can first be analyzed using the BLAST algorithm [85] to find homologous sequences among mammalian species, e.g., human, chimpanzee and mouse. Resulting sequences can be further analyzed for sequence and structure conservation using bioinformatics secondary prediction algorithms. Dynamic programming algorithms such as the Zuker algorithm which are implemented in RNAfold and Mfold calculate for a sequence the thermodynamic optimal secondary structure based on a minimum free energy. These algorithms calculate accurately the optimal secondary structure, they are however not useful for a large-scale application with several sequences due to the high calculation time [86,87,133,134]. A more effective approach represents the Sankoff algorithm which can simultaneously align and fold multiple sequences [88,133,135,136,137]. Programs using the Sankoff algorithm are for instance RNAalifold [88], FOLDALIGN [89] and LocARNA [90], in which a pairwise sequence alignment is generated and subsequently aligned and folded to calculate the optimal conserved secondary structure [135]. However, further extensions of the Sankoff algorithm are more efficient, allowing a faster calculation, e.g., the RNAshapes program has a linear calculation time based on a non-heuristic approach instead of being exponential (Sankoff algorithm) [91,138]. Another useful program for large sequence and structure data sets is the 4SALE algorithm which allows a fast sequence and synchronous secondary analysis including further analyses and manual editing [92].

### 4.3. Functional Interaction Partner Analysis

miRNAs and lncRNAs show enormous clinical potential but currently have some limitations, as exact knowledge about the functional interaction context is necessary. Experimental methods for RNA-target detection range from quantitative proteomic analysis and high throughput experiments such as tissue-specific microarray and RNA-Seq analysis to UV cross-linking immunoprecipitation-high-throughput sequencing (CLIP-Seq) [33,34,83,139]. Such methods are essential for correct identification of interaction partners, but technically challenging, time and cost intensive, e.g., interaction analysis considering the whole interactome is laborious and methods such as CLIP-Seq require a specific target RNA or protein [82,83,115,140]. Over the past decades, several bioinformatics interaction prediction algorithms have been developed which are helpful e.g., for large-scale application and for filtering and pre-selection of candidates for further experimental testing. However, RNA–RNA interaction prediction is challenging due to high combinatorial number of RNA pairs, time complexity of calculating the joint secondary structures of both RNA molecules as well as knowledge of the intra-molecular and inter-molecular base-pairs interactions between both RNA molecules [115]. In the following we will discuss computational databases and prediction algorithm for RNA interactions analysis (extensively reviewed in [33,115,139,141,142,143]).

### 4.4. miRNA Target Prediction Algorithms

miRNAs bind mRNA by seed region matching which can be predicted by bioinformatics algorithms. Numerous prediction algorithms were developed which are mainly based on seed region similarity, but new approaches also include sequence matching combined with structure and/or thermodynamic parameters (folding energy) or target site accessibility [33,34,107,144]. The popular TargetScan algorithm [111] predicts target interactions based on conserved seed region matching, whereas miRanda [105] and PicTar [112] algorithms allow seed region mismatches but include free folding energy, and the PITA algorithm [113] includes target site accessibility for seed interaction prediction [139,145,146,147]. In addition, several other interaction prediction algorithm such as RNAup (target site accessibility [106]) and IntaRNA (seed region and target site accessibility [107]) exist which focus mainly on specific RNA–RNA interactions such as miRNA–mRNA or bacterial small RNAs, but can also be used for other RNA types.

It is worth mentioning that such mRNA target prediction algorithms have some drawbacks and limitations which requires a carefully compliance by users. For instance, most algorithms are not validated by experimental data, they do not include tissue-specific miRNA expression, and are based on different parameters, resulting in a less target overlap between them [33,34,139,144]. Moreover, target predictions often shows large number of false positives, e.g., miRanda has a approximated false-positive rate between 24%–39%, whereas TargetScan shows 22%–31%, PicTar 30% and PITA 20% [113,148,149]. The false positive rate reflects in this case all erroneously predicted target interactions that have not been experimentally validated. Thus, this parameter is of high interest as it gives an approximation of how specific and usable the algorithm is as this is important for computational target interaction prediction especially without available experimental supported results [139].

Beside these facts, several studies highlighted that bioinformatics prediction algorithms are beneficial as an additional tool for experiments [150,151,152]. In this context, studies demonstrated that algorithms using high seed region similarity represent the highest accuracy and overlap of predicted and experimentally validated targets [33,34,150], e.g., TargetScanS (requires perfect complementarity), miRanda and PicTar seem to be the effective methods with a sensitivity nearly 65% with experimentally validated interactions, but miRanda algorithm predicts much more mRNA targets [139,148].

Similarly, Busch et al. (2008) compared several algorithm and demonstrated that IntaRNA shows the highest accuracy, whereas RNAup shows an overall accuracy closest to IntaRNA (average sensitivity of IntaRNA is 0.783, RNAup 0.752) [107]. Therefore, users should use different algorithms and their overlapping targets in combination with experiments to find the best mRNA interaction partners in a functional and tissue-specific manner [33,34,139].

### 4.5. lncRNA–RNA Interaction Prediction Algorithms

Due to the high computational costs and the fact that lncRNAs are a new research field, methods for predicting lncRNA–RNA interactions are limited [115]. To the best of our knowledge, there are currently only two tools available which allow direct lncRNA-RNA interaction prediction. The Rtools pipeline calculates interactions between lncRNA-lncRNA and lncRNA-mRNA considering seed matching and target site accessibility by combining different existing algorithms, e.g., IntaRNA, to reduce the computation complexity. The interactions between lncRNAs and the human transcriptome were calculated, but were also validated using different experimentally RNA–RNA interaction datasets [115]. According to the authors, the pipeline is comparable with existing algorithms but significantly faster, however it has room for further improvements to reach higher accuracy especially for lncRNA interaction predictions [115]. On the other hand, the LncTar software allows calculation of lncRNA-RNA interactions in large-scale datasets [116]. It assumes that the primer-dimer detection is not only important for real-time polymerase chain reaction (PCR) design, but also an important process of base pairing in nature, thus enabling prediction of lncRNA-RNA interactions. Therefore, the software uses a modified exact melting-temperature and primer-dimer prediction algorithms of the PerlPrimer [153] code, a developed platform for real-time PCR primer design [116]. It was demonstrated by the authors that LncTar efficiently predicts lncRNA-RNA interaction partners with highly accuracy, which was further validated by experimentally lncRNA-mRNA interactions curated from the LncRNADisease and NPInter databases [116]. However, it has currently some limitations which need further improvements, e.g., it did not take the stacking base pairs and loop energy for searching the stable joint secondary structures and the RNA tertiary structure into account which are known to play a role in RNA-RNA interactions [116].

miRNAs potentially sponged by lncRNAs can be predicted e.g., using the above described miRanda algorithm [105]. As the RNAup [106] and IntaRNA [107] algorithms can be used for several RNA types, they can also be applied for lncRNA-mRNA interaction prediction. However, they are not efficient for large-scale prediction of lncRNA targets and/or for larger lncRNAs and mRNA molecules, e.g., due to sequence length limitation (IntaRNA ≤ 2 kb, RNAup ≤ 5 kb) [107,116] which requires carefully consideration of their specific features and further steps such as locally software use by users.

For more information and a detailed overview about several *in silico* approaches for functional prediction and mechanistic characterization of lncRNAs please see to the recent review by Signal et al. (2016) [62].

### 4.6. RNA-Protein Interaction Prediction Algorithms

lncRNAs regulate not only RNAs, but also interact with proteins, pointing out the importance of an integrated bioinformatics analysis of potential interaction partners for a tissue and disease-specific functional characterization of lncRNAs. Popular databases such as NPInter [117], the Protein Data Bank (PDB) [118] and Nucleic Acid Database (NDB) [119] include information about the experimental determined structure of proteins and nucleic acids as well as RNA-protein complex assemblies, whereas BioGRID [120] and IntAct [121] contain protein-protein and RNA-protein interactions from several organisms. Furthermore, several approaches combine different databases, thus allowing a comprehensive overview of interactions and further individual analysis. For example, the RPIntDB [82], PRD [122], iMEX [123] and UniProt [124] databases provide functional information and experimentally interactions curated e.g., from BioGRID [120] and IntAct [121], whereas the Protein-RNA Interface Database (PRIDB) [125] collects interactions based on PDB [141]. In addition, the DrumPID database [126] which was developed by our group, focusses especially on the drug-target interaction context combining interaction and pathway data, but also allows organism- or tissue-specific analyses. Additional features include structural and sequence domain analyses of proteins and RNAs which help in detecting functional interaction and recognition binding-sites such as the RNA recognition motif and RNA-binding domain in proteins [126,141,154].

As these databases mainly contain experimentally validated RNA–protein interactions, they are not applicable especially for newly annotated lncRNAs. To support this, several bioinformatics prediction algorithms were developed that focus on the sequence and/or structure by using different machine learning algorithm. For example, the software “fast predictions of RNA and protein interactions and domains at the Center for Genomic Regulation, Barcelona, Catalonia” (catRAPID) [127] performs predictions based on a sequence HMMscan (probabilistic statistical profile Hidden Markov Model (HMM)) using propensities of individual residues from PDB [127,141,142]. The RPISeq software calculates interactions using different machine learning classifiers [82,141,142], whereas Pprint predicts RNA binding sites using evolutionary position-specific scoring matrix (PSSM) information combined with a support vector machine (SVM) [128,155]. The KYG algorithm focuses on the structure and calculates binding regions by applying a position-specific multiple sequence profile on protein-RNA structures from PDB, also without biochemical or functional data [129,155]. Other algorithm like Struct-NB combine sequence and structural-based information of protein-RNA complex interfaces from PDB using a Gaussian Naive Bayes classifiers machine learning algorithm [130], whereas PRINTR calculates interactions using a SVM and PSSM [131,142].

All these algorithms perform high-confidence lncRNA–protein interaction predictions and are helpful to find potential RNA–protein interaction partners [82,141]. Moreover, they reveal a high accuracy, which was validated by independent training datasets including experimentally validated physical interactions [127,129,142,155], e.g., from the NPInter server [117]. Most prominently, catRAPID correctly calculates the interaction of the human lncRNAs XIST and HOTAIR with the Polycomb Repressive Complex 2 (PRC2) but also the interaction between HOTAIR and Suz12 predicted by RPISeq and catRAPID were in agreement with experimental data [82,127,141,142,156].

Nevertheless, these prediction algorithms also have some limitations. For instance, most of them do not consider the tissue and functional specific context of lncRNA-protein interactions and often show large number of interaction predictions [127,141,157]. Moreover, most of them were not systematically validated on general benchmark datasets, depend on different approaches and the prediction outcome is affected by the distance threshold for the interface residue definition [141,155,157]. In addition, several groups evaluated the influence of different machine learning classifiers and found out that the accuracy of sequence-based methods can be increased by using PSSM profiles [128,131,155,158]. For example, Walia et al. (2012) analyzed several sequence and simple structure-based with complex structure-based algorithms and reported that results are comparable between these approaches [155]. However, sequence-based methods using PSSM classifiers achieve comparable results to state-of-the-art structure-based methods, but the latter ones reach higher specificity compared to exclusively sequence-based approaches [155]. Thus, sequence-based approaches can effectively predict RNA–protein interactions, but a higher accuracy can be reached when using PSSM profiles and/or structure-based methods [128,155]. Furthermore, structure-based features also allow identifying substrate-binding clefts and how the RNAs and proteins specifically recognize each other but often show a higher degree of irregularity at the surface compared to non-interface residues. However, they require information on protein–RNA complexes as training templates, which are often limited [131,141,142,155,159].

As parameters and outputs differ between sequence and structure-based approaches, for a large-scale benchmark application and prediction of unknown RNA-protein interactions it is therefore of importance to compare different methods but also how to use them and to interpret the results. In this context, it is essential to have a detailed knowledge about the used features and datasets of each interaction prediction algorithm, the evaluation process and the definition of interface residues, e.g., background data and validation and usage of PSSM profiles [155].

### 4.7. Functional GO and Pathway Enrichment Analysis

Functional enrichment analysis of interaction partners and related regulatory networks is essential for understanding its tissue-specific and functional role and can boost the selection of best candidates for further experimental validation. There exist several databases and software tools for data mining and analysis of large gene list. The most prominent annotation is the Gene Ontology (GO) Consortium project which functionally specifies genes/proteins and their relationships in categories, so-called GO terms, regarding molecular function, cellular component and biological processes (including pathways) in a species-independent manner [93,94]. All annotation data can be downloaded or accessed online from the GO database or through the web-based application database AmiGO which also support a term enrichment analysis for user input lists using Panther [93,94]. The Protein ANalysis THrough Evolutionary Relationships (Panther) classification is a large database collection of protein families that are divided in functional categories using statistical HMMs, allowing functional analysis and classification of large gene lists and/or experimental datasets in significantly enriched ontology and pathway terms [95]. Other popular databases are Reactome, Kyoto Encyclopedia of Genes and Genomes (KEGG) and WikiPathways, enabling also analysis of signaling pathways including tissue analysis, e.g., also from gene lists and/or large-scale expression data sets [96,97,98,160]. Recently, Herwig et al. (2016) developed ConsensusPathDB which allows a functional and network-based characterization of biomolecules from a user input list and/or experimental high-throughput datasets such as RNA-seq [161]. For this, molecular interaction data from 32 different online available repositories from human, mouse and yeast were integrated, in which calculations of statistical significant over-represented and enriched interaction network modules and biochemical pathways are based on different computational analyses.

Limitations and drawbacks are, for instance, large output lists and over-prediction especially from large-scale gene lists. Moreover, results are not filtered for a specific biological process and pathway, and the programs do not consider interactions between genes/proteins and the interaction network in a cell-line and tissue-specific context [33,162,163]. Therefore, an individual collection by users of genes, proteins, processes and signaling pathways associated with lung cancer as well as tissue-specific information will specify the results from the functional enrichment analysis which allows a better functional interpretation in a biological context.

### 4.8. Bioinformatics Tools for Integrated Functional Analysis

There are several bioinformatics tools for integrated functional analysis and interpretation, e.g., co-expression, disease and tissue-specific analysis, enabling to comprehensively understand the functional role of miRNAs and lncRNAs from large input data lists. For example, the starBase web tool deciphers lncRNAs and miRNAs from experimental large-scale CLIP-Seq datasets and tumor samples and provides RNA-protein, miRNA-lncRNA and miRNA-mRNA interactions including further analysis, e.g., biological processes and signaling pathways [100]. Similarly, the lncRNAtor tool is especially designed to investigate and functional understand lncRNAs combining for instance lncRNA expression profiles, RNA-protein interactions and functional enrichment analysis by using RNA-Seq and CLIP-Seq data sets from publicly available databases such as The Cancer Genome Atlas (TCGA), Gene Expression Omnibus (GEO) and ENCODE [132]. Moreover, the Cytoscape software tool allows to visualize and analyze regulatory networks, e.g., regarding functional GO terms and the network topology, co-expression or identification of functional clusters of highly connected interaction partners [99,164,165]. As an example, the Cytoscape plugin ClueGO calculates statistically enriched processes and pathways from a user gene list using GO terms and information from KEGG, WikiPathways and Reactome [166]. Moreover, the ncINetView plugin integrates data from the ncRNA-DB, a database collection of ncRNA interactions from several sources, allowing to search for associated interaction partners and regulatory networks including related biological functions and diseases as well as filtering for a specific diseases [167].

However, current bioinformatics tools only provide information about already known ncRNAs as they mainly analyzed public available large-scale datasets or focus on a specific disease and/or ncRNA class. Nevertheless, for specific analyses, e.g., interaction partners and functional enrichment analysis, there are powerful tools available such as ConsensusPathDB [161] on which further analyses (e.g., promotor, structure as discussed here) can build.

### 4.9. Promoter Analysis

Promoter analysis is an essential step in understanding the complex regulatory effects of ncRNAs, for instance transcriptional regulation of miRNA and lncRNA expression or in case of lncRNAs, also cooperatively working with a TF to guide them to the promoter e.g., to regulate its own transcription in a feedback loop, both helpful in terms of posttranscriptional therapeutically usage. TFs bind to specific transcription factor binding sites (TFBSs) in the promoter that can be bioinformatically represented by a IUPAC (International Union of Pure and Applied Chemistry) consensus nucleotide sequence or a position weight matrices (PWMs), whereas the latter one reflects a better representation by displaying the whole nucleotide distribution for each binding site position (extensively reviewed in [168]). Databases using PWMs are TRANSFAC, JASPAR, Allgen PROMO and MatInspector (implemented in the Genomatix software (Genomatix GmbH, Munich, Germany)). These tools allow not only prediction of putative TFBSs for a given sequence but also additional analysis, e.g., genome-wide and comparative regulatory region analysis [101,102,103,104].

Limitations and drawbacks are for instance, most methods are using different parameters for TFBS detection, they are mostly not based on experimental TFBS profiles and do not consider the tissue and functional context of the TF. Moreover, they are using different output parameters, e.g., dissimilarity threshold which controls how similar a sequence must be to the matrix to be reported as a hit, and do not include multiple statistical testing, indicating a high number of over-predictions. Thus, knowledge about the parameters is essential but also the combination of different prediction software to find overlapping hits. Moreover, analysis regarding the tissue and functional context can improve the detection of potentially functional TFBSs and minimize potential testing candidates which at least need to be further proven by experiments [104].

### 4.10. Automation

As discussed in the previous sections, analyzing ncRNAs is a complicated process involving a lot of different resources, e.g., various databases and prediction tools with different specializations. Especially considering the goal of using circulating miRNA and lncRNA biomarkers in the clinic, solutions for a more automated sample analysis are important. Partial automation has been achieved [115], where multiple prediction algorithms are combined. This already reduces the complexity of the analysis and improves its quality. A similar approach integrates databases, e.g., the online database RNAcentral [108] which provides a unified way to access various previously discussed databases not only using a unified API, but including a comprehensive versioning system which makes the data analysis reproducible through the use of stable identifiers. Nevertheless, complete solutions do not yet exist, but have to be developed using custom scripts or using flexible pipelines like Galaxy [169], Ruffus [170] or Snakemake [171]. Considering the rapidly evolving miRNAs and lncRNA annotations and analysis tools, reproducibility and versioning of the analysis pipeline is important. Pipeline specific [172] as well as generic approaches using Docker [173] have been proposed.

## 5. Conclusions and Future Directions

**Figure 3:**
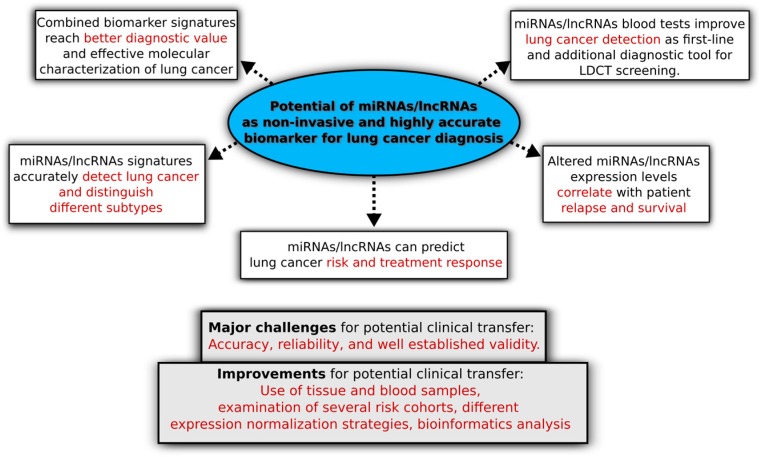
Schematic overview of key points of the potential of miRNAs and lncRNAs as non-invasive biomarker in lung cancer diagnosis. The Figure summarizes key points of the potential of miRNAs and lncRNAs as non-invasive biomarker in lung cancer diagnosis, current challenges and useful improvements for a clinical transfer in the future.

miRNAs and lncRNAs have a high potential working as non-invasive biomarker in lung cancer diagnosis (see Figure 3 for a summary of key points presented in this review). First large-scale blood biomarker signature studies based on miRNAs and lncRNAs show promising clinical results for NSCLC early diagnosis. However, studies to understand the implication and potential of such circulating biomarker signatures in lung cancer have just begun. There are major challenges for the transfer to the clinic, e.g., accuracy, reliability, and well established validity. Thus, further investigations and validation studies are required, identifying highly sensitive tests for efficient RNA-based lung cancer diagnostics. Regarding their functional characterization, experimental methods are technical challenging and laborious, but can be supported by integrated bioinformatics analysis for filtering and pre-selection of experimental candidates considering the tissue and functional interaction context. Especially lncRNA characterization is challenging and functional understanding of their role is limited which requires further studies regarding the diagnostic potential. Blood-based tests must be highly accurate and face substantial hurdles [15,22,174], however, such biomarker signatures have enormous potential as a first-line diagnostic tool. Despite its proven potential to reduce false-positive rates of LDCT screenings as an additional diagnostic tool, further refinement and evaluation of blood signatures are necessary. In this context, the use of tissue (e.g., from surgical NSCLC biopsies and/or GEO) and blood samples, examination of several cohorts (e.g., low- and high-risk lung cancer patients), expression normalization and analysis using different normalization strategies as well as bioinformatics meta-analysis will be essential to improve lung cancer diagnostics and application in the clinic in future. This will not only refine the blood biomarker signature but can also reduce the complexity of such blood tests, e.g., due to the identification of a combination of two or three differentially expressed miRNAs and lncRNAs. All this will contribute to better accuracy and less costs, resulting in higher number of correct and early diagnosed individuals as well as the reduction of unnecessary invasive diagnostics. The vision for the future is that circulating miRNAs and lncRNAs based blood tests can be transferred to the clinic for a better clinical management of lung cancer resulting in a better patient survival.

## Figures and Tables

**Table 1:** Table summarizing the different lung cancer miRNA studies.

Study (Ref.)	Patient Cohort	Important Reported Findings
Tang et al. (2013) [37]	Training set: 62 patients/60 healthy smokers Test set: 34 malignant tumor patients, 30 patients with benign pulmonary nodules, 32 healthy smokers	Plasma miRNA-21, miRNA-145 and miRNA-155 have strong potential as novel noninvasive biomarkers for early detection of lung cancer
Yu et al. (2008) [42]	112 NSCLC patients: AC (55), SQ (50), others (7)	five-miRNA signature (miRNA-221, let-7a, miRNA-137, miRNA-372, miRNA-182 *) for NSCLC treatment prediction outcome
Yanaihara et al. (2006) [43]	AC (65), AC normal (65); SQ (39), SQ normal (39) Stage classification: AC: stage I (41), stage II (8), stages III and IV (16); SQ: stage I (24), stage II (9), stages III and IV (6)	miRNA-155 and let-7a-2 correlates with poor survival; miRNA-205, miRNA-99b, miRNA-203, miRNA-202, miRNA-102 and miRNA-204-prec differently expressed in AC and SQ; miRNA-21, miRNA-191, miRNA-155, miRNA-210, miRNA-126 * and miRNA-224 differentiate AC and SQ
Geng et al. (2014) [39]	Training set: 25 NSCLC patients: AC (8), SQ (13), others (4); stage: I (9), II (16); 25 healthy controls Test sample: 126 NSCLC patients: AC (45), SQ (64), others (17); stage: I (54), II (72); 42 NCPD (non-cancerous pulmonary disease) patients; 60 healthy controls	Plasma miRNA-20a, miRNA-145, miRNA-21, miRNA-223 and miRNA-221 as potential biomarkers in early-stage NSCLC
Zhu et al. (2016) [40]	112 NSCLC patients: AC (90), SQ (22); lymph node metastasis: negative (95), positive (17); stage: 0 (0), IA, IB (82), IIA, IIB (15), IIIA, IIIB (10); 104 controls (20 current healthy smokers, 23 pneumonia patients, 21 gastric cancer patients, 40 healthy controls)	Serum miRNA-182, miRNA-183, miRNA-210 and miRNA-126 levels serve as a diagnostic biomarker for NSCLC early detection; serum levels distinguish NSCLC or early-stage NSCLC from current smokers without lung cancer and pneumonia or gastric cancer patients
Bjaanaes et al. (2014) [41]	154 resected AC: stage: IA (45), IB (46), IIA (24), IIB (12), IIIA (26), IV (1); *EGFR* status: mutated (22), wt (130), not tested (2); *KRAS* status: mutated (52), wt (96), not tested (6); 20 normal tissue samples; independent cohort of 103 lung cancer patients	129 significantly differentially expressed miRNAs in AC compared with normal lung tissue; 17 differentially expressed miRNAs between EGFR-mutated and EGFR wildtype AC; miRNA-9, miRNA-21, miRNA-126, miRNA-133a and miRNA-500a deregulated in AC compared with normal lung tissue
Saito et al. (2011) [44]	317 AC patient tissues: stage: I (220), II (76), III (21)	miRNA-21 is associated with disease progression and survival in stage I AC
Capodanno et al. (2013) [45]	80 NSCLC patients: *EGFR* status: Wt: AC (32), SQ (20), LCC and other (3); stage: I (9), II (20), III (13), IV (3); mutated: AC (22), SQ (0), LCC and other (1); stage: I (3), II (11), III (1), IV (3) *KRAS* status: Wt: AC (39), SQ (20), LCC and other (3); stage: I (9), II (27), III (9), IV (4); mutated: AC (15), SQ (0), LCC and other (1); stage: I (3), II (4), III (5), IV (2)	let-7g and miRNA-21 combined with KRAS mutational status are useful biomarkers for NSCLC patients
Du et al. (2010) [5]	19 lung cancer cell lines: 7 NSCLC (2 AC, 3 SQ, 2 other); 9 SCLC; 3 immortalized normal	41 out of 136 differentially expressed miRNAs distinguish NSCLC and SCLC (e.g., miRNA-17-5p, miRNA-135, miRNA-103, miRNA-107, miRNA-301 and miRNA-338 altered in SCLC relative to NSCLC); miRNA-29a/b/c, miRNA-24, miRNA-21 and miRNA-221/222 down-regulated in SCLC
Lee et al. (2011) [46]	26 NSCLC cell lines, 14 SCLC cell lines, 31 SCLC tumors	miRNA-21, miRNA-29b, miRNA-34a/b/c, miRNA-155 and let-7a not related to SCLC patients
Landi et al. (2010) [30]	290 tissue samples: AC (165): stage: I (65), II (43), III (46), IV (11); SQ (125): stage: I (52), II (42), III (30), IV (1)	34 miRNAs differentiate AC from SQ in male smoker patients; five-miRNA signature predicts survival for SQ
Lebanony et al. (2009) [47]	Training set: 122 AC and SQ; 47 NSCLC FFPE samples Test set: 79 blinded cohort of NSCLC FFPE samples	miRNA-205 expression distinguishes SQ from AC
Bishop et al. (2010) [48]	102 resected NSCLC: AC (50): grades: well (9), moderate (24), poor (17); SQ (52): grades: well (2), moderate (35), poor (15); 21 preoperative biopsies/aspirates	miRNAs such as miRNA-205 are reliable to classify NSCLC
Montani et al. (2015) [14]	COSMOS study with high-risk individuals (n = 1115): Calibration Set: lung cancer (12): stage: I (11), II-III (1); lung cancer deaths (1); no lung cancer (12) Validation Set: lung cancer (36): stage: I (31), II-III (5); lung cancer deaths (3); no lung cancer (972); PN+COPD (81) Specificity Set: no lung cancer (83); PN + COPD + NOD (78); Benign (5) All (CT screening): lung cancer (48): stage: I (42), II-III (6); lung cancer deaths (4); no lung cancer (1067); PN + COPD + NOD (159); Benign (5)	miR-Test using 13 miRNAs (miRNA-92a-3p, miRNA-30b-5p, miRNA-191-5p, miRNA-484, miRNA-328-3p, miRNA-30c-5p, miRNA-374a-5p, let-7d-5p, miRNA-331-3p, miRNA-29a-3p, miRNA-148a-3p, miRNA-223-3p, miRNA-140-5p) represent a useful tool for lung cancer screening in high-risk individuals
Sozzi et al. (2014) [13]	MILD trial study: 939 plasma samples: no lung cancer (870); lung cancer (69): stage: I (37); II to III (12); IV (19)	Plasma-based miRNA signatures from patients in two independent LDCT screening studies of 24 circulating miRNAs has diagnostic and prognostic performance
Hennessey et al. (2012) [49])	Phase I/II serum biomarker study: Training set: 30 NSCLC patients: AC (20), SQ (10); stage: I (10), II (9), III (10), IV (0); 20 healthy controls Test set: 55 NSCLC patients: AC (30), SQ (25); stage: I (33), II (13), III (7), IV (2); 75 healthy controls	Combination of miRNA-15b and miRNA-27b discriminate NSCLC from healthy controls
Markou et al. (2013) [50]	59 resected NSCLC and adjacent normal tissue: Training set: 19 tumor/normal tissues Test set: 40 tumor: AC (16), SCC (21), Other (3); stage: I (11), II-IV (29); lymph node: negative (23), positive (17); plasma samples from 37 NSCLC patients and 28 healthy donors	8 circulating plasma miRNAs (miRNA-21, miRNA-30d, miRNA-451, miRNA-10a, miRNA-30e-5p, miRNA-126 *, miRNA-126, miRNA-145) were differential expressed in NSCLC; miRNA-520d, miRNA-489, miRNA-181b, miRNA-513, miRNA-26b, miRNA-189 and miRNA-520e were differentially expressed between AC and SQ
Wang et al. (2011) [51]	88 NSCLC patients: AC (37), SQ (21), other (30); stage: I–II (47), III (41); lymph node metastasis: No (53), Yes (35); 17 healthy individual	Serum miRNA-21 expression useful as a prognostic marker for NSCLC patients

* Indicates the antisense miRNA product.

**Table 2:** Table summarizing the different lung cancer lncRNA studies.

Study (Ref.)	Patient Cohort	Important Reported Findings
Zhu et al. (2015) [29]	Meta-Analysis: 8 studies with 845 patients: NSCLC (2), colorectal cancer (1), gastric cancer (1), pancreatic cancer (2), clear cell renal cell carcinoma (1), osteosarcoma (1)	MALAT-1 serve as a molecular marker for cancer metastasis and prognosis
Ji et al. (2003) [68]	NSCLC patients (70): AC (26), SQ (34), LCC (10); stage: I (37), II (13), IIIA (20)	MALAT-1 and thymosin beta4 predict metastasis and survival in early-stage non-small cell lung cancer
Weber et al. (2013) [22]	45 NSCLC patients: AC (21): Stage: I/II (0),III/IV (21); SQ (24): I/II (3), III/IV (21); 25 controls	MALAT1 as complementary diagnostic biomarker in NSCLC
Yao et al. (2012) [12]	65 NSCLC patient serum: biopanning stage: tissue and serum samples (25): AC (12), SQ (12), LCC (1); stage: I (6), II (5), III (9), IV (5) identification stage: NSCLC serum samples (40): AC (17), SQ (22), LCC (1); stage: I (11), II (8), III (17), IV (4); 41 normal controls	A four serum biomarker SMOX, NOLC1, MALAT1 and HMMR show a high diagnostic accuracy for detecting early stage NSCLC
Qiu et al. (2014) [69]	NSCLC tissues/paired adjacent normal tissues	CCAT2 is an AC-specific lncRNA and promotes invasion of NSCLC; biomarker for lymph node metastasis.
Chen et al. (2016) [70]	SCLC tissues and cell lines	CCAT2 serves as an oncogenic lncRNA, and an independent unfavorable prognostic factor in SCLC patients
Liu et al. (2013) [71]	Tissues from 42 NSCLC/adjacent non-tumor lung patients: stage: I/II (25), III/IV (17); 4 NSCLC cell lines: AC (3; A549, SPC-A1, NCI-H1975); SQ (1; SK-MES-1); normal human bronchial epithelial cell line (1; 16HBE)	HOTAIR represent diagnostic biomarker of poor prognosis in NSCLC
Yang et al. (2013) [72]	A549 cells and cisplatin resistant A549/CDDP cells (microarray profiling of mRNAs, lncRNAs and miRNAs)	8 mRNAs (BMP4, CTSB, NKD2, BAG1, TGFB1, EGFR, JUN, CUL2), 8 lncRNAs (AK123263, CES1P1-001, RP3-508I15.14, AK126698, TP53TG1, AC090952.4.1, uc003bgl.1, NCRNA00210) and 5 miRNAs (miRNA-17, miRNA-21, let-7i, miRNA-138, miRNA-194) potentially play a key role in cisplatin resistance; AK126698 appears to confer cisplatin resistance by targeting the Wnt pathway
Sui et al. (2016) [73]	465 AC patient RNA sequencing profiles (from TCGA); 53 AC patients	Correlation of AFAP1-AS1 and LINC00472 as potential biomarkers for diagnosis and prognosis
Tantai et al. (2015) [74]	64 NSCLC tissues/matched adjacent non-tumor patient tissues; stage: I (15), II/III (17)	Combination of XIST and HIF1A-AS1 had a higher positive diagnostic efficiency of NSCLC patient screening
Gong et al. (2016) [75]	498 lung cancer patients (467 patients at least two cycles of platinum-based chemotherapy); 213 healthy controls	HOTTIP, CCAT2, H19, HOTAIR, MALAT1 and ANRIL potential clinical biomarkers to predict lung cancer risk and platinum-based chemotherapy response
Yuan et al. (2016) [76]	Meta-analysis of eight published GWAS datasets with 17,153 cases and 239,337 controls	SNP rs114020893 of NEXN-AS1 at 1p31.1 may contribute to lung cancer susceptibility
Yang et al. (2014) [6]	5 NSCLC gene expression datasets from GEO: Training set: GSE27262, GSE19804, GSE19188 and GSE30219 Test set: GSE18842	47 lncRNAs differentially expressed in NSCLC; 19 lncRNAs differed in expression between SCC and AC
White et al. (2014) [7]	Three lung RNA-Seq datasets: 72 AC/adjacent normal pairs 55 AC/adjacent normal pairs + 243 unmatched tumors from TCGA 34 SQ/adjacent normal pairs + 163 unmatched tumors from TCGA	463 and 315 up- and down-regulated lncRNA in AC tumors relative to SQ; 27 lncRNAs differentially expressed between AC and SQ
Zhang et al. (2015) [2]	AC and SQ microarray	1646 differentially expressed lncRNA LINC01133 showed the largest up-regulation in SQ but not in AC
Wei et al. (2016) [1]	Paired tissue samples of RNA sequencing or microarray data from TCGA and GEO	lncRNA expression is different in AC and SQ knockdown of the up-regulated lncRNA AFAP1-AS1 and LINC00511 impaired AC cell proliferation; knockdown of PVT1 inhibited SQ cell growth four 6-lncRNAs signature expression patterns were found to be significantly associated with AC and SQ patient overall and progression-free survival

**Table 3:** Databases and software packages for ncRNA research.

Tool (Ref.)	Purpose	Website
Rfam [84]	ncRNA database	http://rfam.xfam.org/
Ensembl	genome browser	http://www.ensembl.org/
UCSC	genome browser	https://genome.ucsc.edu/
BLAST [85]	Sequence analysis	http://blast.ncbi.nlm.nih.gov/Blast.cgi
RNAfold [86]	Folding prediction	http://rna.tbi.univie.ac.at/cgi-bin/RNAfold.cgi
Mfold [87]	Folding prediction	http://unafold.rna.albany.edu/?q=mfold
RNAalifold [88]	Folding prediction	http://rna.tbi.univie.ac.at/cgi-bin/RNAalifold.cgi
FOLDALIGN [89]	Folding prediction	http://rth.dk/resources/foldalign/server/index.html
LocARNA [90]	Folding prediction	http://rna.informatik.uni-freiburg.de/LocARNA/Input.jsp
RNAshapes [91]	Folding prediction	https://bibiserv2.cebitec.uni-bielefeld.de/rnashapes
4SALE [92]	Folding prediction	http://4sale.bioapps.biozentrum.uni-wuerzburg.de/
GO database [93]	Functional classification	http://geneontology.org/page/go-database
AmiGO [94]	Functional analysis	http://amigo.geneontology.org/amigo
Panther [95]	Functional analysis	http://www.pantherdb.org/
Reactome [96]	Interactions/pathways	http://www.reactome.org/
KEGG [97]	Interactions/pathways	http://www.genome.jp/kegg/
WikiPathways [98]	Interactions/pathways	http://wikipathways.org/index.php/WikiPathways
Cytoscape [99]	Visualization/Functional analysis	http://www.cytoscape.org/
starBase v2.0 [100]	Functions/Interactions/networks	http://starbase.sysu.edu.cn/
TRANSFAC [101]	Promotor analysis	http://www.gene-regulation.com/pub/databases.html
JASPAR [102]	Promotor analysis	http://jaspar.genereg.net/
Allgen PROMO [103]	Promotor analysis	http://alggen.lsi.upc.es/cgi-bin/promo_v3/promo/promoinit.cgi?dirDB=TF_8.3
MatInspector [104]	Promotor analysis	https://www.genomatix.de/online_help/help_matinspector/matinspector_help.html
miRanda [105]	Target prediction	http://www.microrna.org/microrna/home.do
RNAup [106]	Target prediction	http://rna.tbi.univie.ac.at/cgi-bin/RNAup.cgi
IntaRNA [107]	Target prediction	http://rna.informatik.uni-freiburg.de/IntaRNA/Input.jsp
RNAcentral [108]	nRNA sequence database	http://rnacentral.org/

**Table 4:** Databases and software packages for miRNA research.

Tool (Ref.)	Purpose	Website
miRBase [109]	miRNA database	http://www.mirbase.org/
MiR2Disease [110]	Interactions/pathways	http://www.mir2disease.org/
TargetScan [111]	Target prediction	http://www.targetscan.org/vert_71/
PicTar [112]	Target prediction	http://www.pictar.org/
PITA [113]	Target prediction	https://genie.weizmann.ac.il/pubs/mir07/mir07_data.html

**Table 5:** Databases and software packages for lncRNA research.

Tool (Ref.)	Purpose	Website
LncRBase [60]	lncRNA database	http://bicresources.jcbose.ac.in/zhumur/lncrbase/
LNCipedia [59]	lncRNA database	http://www.lncipedia.org/
LncRNADisease [114]	Interactions/pathways	http://www.cuilab.cn/lncrnadisease
Rtools [115]	Interactions/pathways	http://rtools.cbrc.jp/cgi-bin/RNARNA/index.pl
LncTar [116]	Interactions	http://www.cuilab.cn/lnctar
NPInter [117]	Interactions	http://www.bioinfo.org/NPInter/
PDB [118]	Interactions	http://www.rcsb.org/pdb/home/home.do
NDB [119]	Interactions	http://ndbserver.rutgers.edu/
BioGRID [120]	Interactions	https://thebiogrid.org/
IntAct [121]	Interactions	http://www.ebi.ac.uk/intact/
PRD [122]	Interactions	http://pri.hgc.jp/
RPIntDB [82]	Interactions	http://pridb.gdcb.iastate.edu/RPISeq/RPIntDB.html
iMEX [123]	Interactions	http://www.imexconsortium.org/
UniProt [124]	Interactions	http://www.uniprot.org/
PRIDB [125]	Interactions	http://pridb.gdcb.iastate.edu/
DrumPID [126]	Interactions/pathways	http://drumpid.bioapps.biozentrum.uni-wuerzburg.de/compounds/index.php
catRAPID [127]	Interaction prediction	http://s.tartaglialab.com/page/catrapid_group
RPISeq [82]	Interaction prediction	http://pridb.gdcb.iastate.edu/RPISeq/
Pprint [128]	Interaction prediction	http://www.imtech.res.in/raghava/pprint/
KYG [129]	Interaction prediction	http://cib.cf.ocha.ac.jp/KYG/
Struct-NB [130]	Interaction prediction	http://www.public.iastate.edu/~ftowfic
PRINTR [131]	Interaction prediction	http://210.42.106.80/printr/
lncRNAtor [132]	Functions/Interactions/networks	http://lncrnator.ewha.ac.kr/index.htm
